# Aboveground and Belowground Herbivores Synergistically Induce Volatile Organic Sulfur Compound Emissions from Shoots but Not from Roots

**DOI:** 10.1007/s10886-015-0601-y

**Published:** 2015-07-21

**Authors:** Holger Danner, Phil Brown, Eric A. Cator, Frans J. M. Harren, Nicole M. van Dam, Simona M. Cristescu

**Affiliations:** Department of Molecular Interaction Ecology, Institute for Water and Wetland Research (IWWR), Radboud University, PO Box 9010, 6500 GL Nijmegen, The Netherlands; Life Science Trace Gas Facility, Institute for Molecules and Materials, Radboud University, Nijmegen, The Netherlands; Department of Applied Stochastics, Institute for Mathematics, Astrophysics and Particle Physics, Radboud University, Nijmegen, The Netherlands; German Centre for Integrative Biodiversity Research (iDiv) Halle-Jena-Leipzig, Deutscher Platz 5e, 04103 Leipzig, Germany; Institute of Ecology, Friedrich Schiller University Jena, Dornburger-Str. 159, 07743 Jena, Germany

**Keywords:** Aboveground-belowground interactions, Trace gas analysis, Herbivore induced plant responses, Volatile organic compound (VOC), Proton transfer reaction mass spectrometry (PTR-MS), Sulfur-containing compounds

## Abstract

Studies on aboveground (AG) plant organs have shown that volatile organic compound (VOC) emissions differ between simultaneous attack by herbivores and single herbivore attack. There is growing evidence that interactive effects of simultaneous herbivory also occur across the root-shoot interface. In our study, *Brassica rapa* roots were infested with root fly larvae (*Delia radicum*) and the shoots infested with *Pieris brassicae*, either singly or simultaneously, to study these root-shoot interactions. As an analytical platform, we used Proton Transfer Reaction Mass Spectrometry (PTR-MS) to investigate VOCs over a 3 day time period. Our set-up allowed us to monitor root and shoot emissions concurrently on the same plant. Focus was placed on the sulfur-containing compounds; methanethiol, dimethylsulfide (DMS), and dimethyldisulfide (DMDS), because these compounds previously have been shown to be biologically active in the interactions of *Brassica* plants, herbivores, parasitoids, and predators, yet have received relatively little attention. The shoots of plants simultaneously infested with AG and belowground (BG) herbivores emitted higher levels of sulfur-containing compounds than plants with a single herbivore species present. In contrast, the emission of sulfur VOCs from the plant roots increased as a consequence of root herbivory, independent of the presence of an AG herbivore. The onset of root emissions was more rapid after damage than the onset of shoot emissions. The shoots of double infested plants also emitted higher levels of methanol. Thus, interactive effects of root and shoot herbivores exhibit more strongly in the VOC emissions from the shoots than from the roots, implying the involvement of specific signaling interactions.

**Electronic supplementary material:**

The online version of this article (doi:10.1007/s10886-015-0601-y) contains supplementary material, which is available to authorized users.

## Introduction

Upon herbivore attack, plants employ an arsenal of defense responses, including the production of volatile organic compounds (VOCs). These herbivore-induced plant volatiles have long been recognized as an indirect defense mechanism against herbivores by attracting natural enemies of the herbivores (Price et al. 1980). Many studies have investigated plant volatile mediated interactions aboveground (AG) (reviewed in Heil 2008; Mumm and Dicke 2010). Volatile organic compound emissions also are active in belowground (BG) tri-trophic interactions (Neveu et al. 2002; Rasmann et al. 2005; van Tol et al. 2001). As a consequence, the induction of VOCs by BG herbivores and their role in attracting natural enemies to the plant has gained more attention (Ali et al. 2010; Crespo et al. 2012; Ferry et al. 2007; Pierre et al. 2012; Rasmann et al. 2011; van Dam et al. 2012)

Most studies on induced indirect defenses, either above or below ground, have investigated natural enemy responses and VOCs of plants damaged by a single herbivore species. In nature, plants are frequently challenged by attacks from multiple species, either simultaneously or sequentially. Studies on multiple herbivores and their effect on AG multi-trophic interactions have shown that simultaneous attacks by different species of herbivores result in induced responses that differ significantly from those seen after single herbivory (Dicke et al. 2009). As a consequence, the interactions of multiple species can affect the behavior of higher trophic levels, and thus may compromise the effectiveness of VOCs as indirect defenses (de Rijk et al. 2013; Soler et al. 2005). When the VOC profile is changed by a non-host present on the same plant, the searching efficiency and oviposition rates of parasitoids using VOCs as cues to find their host may be reduced (de Rijk et al. 2013). There is growing evidence that such interactive effects via herbivore induced responses also occur across the root-shoot interface (Bezemer and van Dam 2005; Soler et al. 2005, 2007, 2013; van Dam and Heil 2011). Thus, AG and BG herbivores and their natural enemies form a complex network of interacting species connected via systemically induced plant responses (Bezemer and van Dam 2005; Wardle et al. 2004).

One of the best studied AG-BG multitrophic complexes consists of *Brassica* spp infested with the shoot herbivore *Pieris brassicae* (large cabbage white) and the root herbivore *Delia radicum* (cabbage root fly), and their respective parasitoids *Cotesia glomerata* and *Trybliographa rapae*. Both shoot feeding by *Pieris* spp., as well as root damage by *D. radicum* larvae, elicit the production of a wide variety of VOCs in the headspace of the plant (Geervliet et al. 1997; Pierre et al. 2011b). These herbivore-induced VOCs have been shown experimentally to serve as cues for both AG and BG parasitoids. Sulfur-containing compounds may play a role in these interactions. Specifically, root fly damage causes an induced emission of methanethiol, dimethylsulfide (DMS), and dimethyldisulfide (DMDS) from roots (Crespo et al. 2012; van Dam et al. 2012). Staphylinid beetles, the main predators of *D. radicum*, are attracted by DMDS to *Delia*-infested plants (Ferry et al. 2007). It has been suggested that DMDS also serves as a signal for AG foraging *C. glomerata* parasitoids. They use it as a cue to avoid ovipositing in caterpillars feeding on the shoots of root fly infested plants on which the performance of their offspring is reduced (Soler et al. 2005). Isothiocyanates (ITCs), the reaction products of glucosinolates and myrosinase, are another class of sulfur-containing compounds typical for *Brassicaceae* and important in this system. They are attractive to both the cabbage root fly (Kostal 1992) and to specialist AG herbivores searching for *Brassica* hosts (Bruce 2014). In addition, ITCs also may serve as a cue to parasitoids specialized on *Brassica* herbivores (Mumm and Dicke 2010; Pope et al. 2008). Upon formation after damage, ITCs may be partly converted into sulfides by the enzyme TMT, supposedly reducing autotoxicity of these reactive compounds (Attieh et al. 2000). Thus, sulfur-containing VOCs are a biologically relevant segment of the infochemical network of *Brassica* species. However, compared to other herbivore-induced VOCs, such as terpenes or green leaf volatiles (see Mumm et al. 2008), they have received relatively little attention so far.

The present study examines how feeding by *P. brassicae* on the shoot or root feeding by *D. radicum*, alone and in combination, influences the emission of sulfur-containing VOCs emitted from wild *Brassica rapa* plants. Previous studies on the herbivore-induced VOC emissions of *Brassica* species have either analyzed BG induced responses only (Crespo et al. 2012; van Dam et al. 2012), or the VOCs were collected from the total headspace of roots and shoots together (Geervliet et al. 1997; Pierre et al. 2011b; Soler et al. 2007). Instead, our experiment used a set-up that allowed root and shoot emissions to be monitored separately, yet concurrently on the same plant. Thus, we could assess whether the changes in the behavior of AG or BG parasitoids towards double infested plants are due to local or to systemic induced responses. Moreover, interactive effects on the induced response due to herbivory on the other plant organ could be identified by comparing organ-specific responses of single and double infested plants.

Time-resolved measurements may provide a better insight into the occurrence of diurnal patterns and other temporal variations in herbivore-induced VOC emissions than conventional VOC collections. A more detailed knowledge of such temporal patterns may contribute to a better understanding of the ecological role of VOCs, especially regarding variation in behavioral responses of organisms, such as parasitoids, over the course of the plant-herbivore interaction (see Mathur et al. 2013). Here, we used Proton Transfer Reaction Mass Spectrometry (PTR-MS) to simultaneously investigate VOCs emitted from roots and shoots over 3 to 4 day time periods. The instrument uses H_3_O^+^ to chemically ionize VOCs, which are subsequently analyzed by a quadrupole mass spectrometer according to their mass-to-charge ratio, *m/z* (usually their molecular mass plus 1; Boamfa et al. 2004). This technique allows for the monitoring of VOC emissions, without any pre-analytical steps, at and below ppbv levels (ppbv, parts per billion per volume). The plant organs were enclosed by cuvettes and their emissions entered directly into the instrument, making possible the study of VOC emissions online. A detailed description of the technique for the analyses of plant VOCs has been published previously (Danner et al. 2012). In addition to sulfur-containing VOCs, we also monitored methanol (*m/z* 33) emissions. Studies on other plant species have shown that methanol emissions from the shoot increase strongly upon AG herbivory (Penuelas et al. 2014; von Dahl et al. 2006). However, it is as yet unknown whether root herbivory would have a similar effect.

With our experimental setup, we measured local, systemic, and interactive effects of single and dual herbivory on sulfur-containing VOCs emitted from roots and shoots of *Brassica rapa*. Based on previous observations of changes in the whole plant head space and altered parasitoid behavior towards double infested plants, (Pierre et al. 2011a, b; Soler et al. 2007), we postulated that double infestations would enhance the emissions of sulfur compounds from both compartments. Our results provide insights into the potential mechanisms, such as the direction of defense signaling between roots and shoots on single and double infested plants.

## Methods and Materials

### Plants and Insect Rearing

**Fig. 1:**
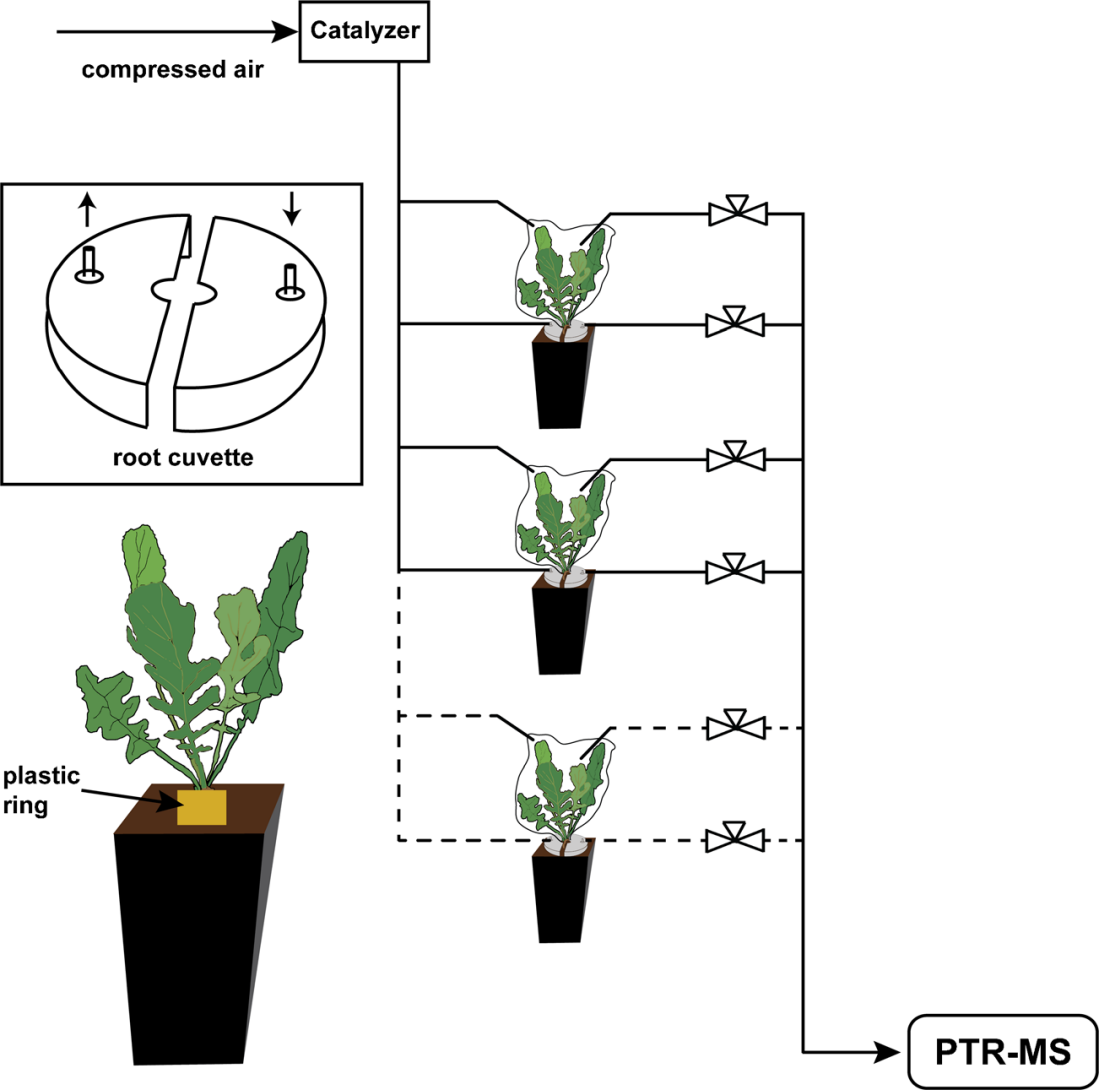
Schematic overview of the set-up for measuring root and shoot emitted volatile organic compounds (VOCs). A constant flow of 2.25 l/h hydrocarbon-free air was applied to flush the headspace of root-cuvettes and PET oven bags which served as the cuvettes for the aboveground (AG) plant parts. *Inset 1*: Schematic drawing of the root cuvette. The gap between the two parts was closed with a solvent-free rubber-based sealant. *Inset 2*: The plants potted with an additional plastic ring (removed before measurements) to give access to the root-stem interface for attaching root cuvettes and oven bags

Seeds were provided by Tom de Jong of Leiden University (The Netherlands) and originated from a wild population of *Brassica rapa* (Maarsen, The Netherlands) in 2009. Seedlings were obtained by germination onto glass beads, held in plastic containers closed with a transparent plastic lid and kept at constant temperature and humidity (24 °C, 70% rel. humidity) under long day conditions (16 h:8 h L:D cycle) in a climate chamber for 1 week (SNIJDERS Labs, Tilburg, The Netherlands). Seedlings subsequently were transferred to 2.2 l pots, (11 × 11 × 21.5 cm) filled with potting soil (Lentse Potgrond n°4, Horticoop, Bleiswijk, The Netherlands) and covered with a 3 cm layer of plain river sand, which facilitated the assessment of root damage and the access to root fly larvae and pupae. Seedlings were placed in the center of a sand-filled plastic foil ring, (height 3 cm) placed on top of the sand layer. The ring including the sand was removed before the VOC collections so that shoot enclosures as well as root cuvettes could be easily assembled to the plant’s stem-root interface (Fig. 1).

Plants were grown in an insect-free greenhouse for 4 weeks maintaining the L:D cycle at 16:8 h with high-pressure sodium lamps (Philips, Eindhoven,The Netherlands) when photosynthetically active radiation was lower than 250 μmol · m^−2^ · s^−1^, until they had at least six fully developed leaves. Root fly larvae were obtained from our own rearing, maintained on turnips as described in (Neveu et al. 2002). Larvae in the second and third instar (L2/L3) were used. *Pieris brassicae* caterpillars were obtained as eggs from a culture at Wageningen University (The Netherlands) and reared on broccoli plants until L2/L3 stage.

### Plant Treatments and Herbivore Infestations

Plants were infested either belowground with root fly larvae (*Delia radicum*), aboveground with *Pieris brassicae* caterpillars, or below- and aboveground with both herbivores. Control plants remained insect-free. Infestations with *D. radicum* consisted of 6–8 L2/L3 larvae depending on the diameter of the visible upper part of the main root. Infestations with *P. brassicae* consisted of 15–20 L2/L3 caterpillars depending on the size of the plant leaves to obtain damage levels between 5 and 10% after 3 days of continuous feeding by the herbivores. Immediately after the plants were infested with their respective herbivores, they were prepared for sampling as described in the next section. Plants were harvested immediately after the measurements had finished (approx. 86 h after infestation with herbivores). Shoot herbivory was estimated by visual inspection and scored in 5 different classes (0–1, 1–5, 5–10, 10–20, 20–50%). For assessing root damage, we carefully washed the roots, and noted whether and to what extent the roots were damaged by larval feeding, based on the total area of brown feeding trenches on the surface of the main root. We distinguished four categories: no visible damage; visible, darkened feeding trenches; substantial feeding trenches; cut-off root. After assessing the damage, roots and shoots were flash-frozen immediately in liquid nitrogen and subsequently freeze-dried to determine dry weights.

### Proton Transfer Reaction Mass Spectrometry (PTR-MS)

Time-resolved measurements of VOCs were accomplished with an in-house constructed PTR-MS described in detail in (Boamfa et al. 2004). The instrument was used in Selective Ion Monitoring (SIM) mode, with settings optimized for measuring sulfur compounds (Samudrala et al. 2015). The calibration of the system was performed with a standard gas mixture containing seven pure compounds (acetaldehyde, acetone, isoprene, benzene, toluene, xylene, and α-pinene) in the range from 32 to 136 Da in concentrations of 1 ppmv (+/− 5%; Linde, Dieren, The Netherlands) in nitrogen. As this study focused on sulfides, which were not contained in the standard gas mixture, separate standards were prepared for methanethiol (*m/z* 49), dimethylsulfide (*m/z* 63, DMS), and dimethyldisulfide (*m/z* 95, DMDS). All standards were diluted with hydrocarbon-free air to obtain a calibration curve with at least five points in the range between 100 and 600 ppbv (ppbv – parts per billion per volume), which covers plant emission rates (Crespo et al. 2012; Danner et al. 2012; van Dam et al. 2012). In this way, we obtained calibration factors for these compounds and used them to convert the ion intensities into gas mixing ratios (ppbv, parts per billion per volume).

Roots and shoots from several plants were measured simultaneously by using a valve system with 17 channels. An Arduino MEGA board (Arduino, Italy) together with a custom made program written in the Arduino Software environment controlled the valves to obtain consecutive cyclic measurements of all replicates and treatments. Each cuvette was sampled in turn for an average of 450 sec with a mean dwell time of 0.32 sec for each *m/z*. In this way, VOC emissions from AG to BG organs of eight plants could be measured for three consecutive days. We performed four experiments, each with two replicates for each of the four treatments. After recording the emissions of specific masses in selective ion monitoring mode, the resulting data were exported to a text file and further processed with a custom-written script in R (R Core Team 2014).

The AG plant parts were enclosed in PET oven bags (45 × 55 cm, Dumil®, ITH Complast, Zwijndrecht, The Netherlands), tightly closed below the rosette leaves with a cable tie. A flow of clean air (2.25 l/h) was continuously maintained through the oven bag during the measurement period. Headspace samples for PTR-MS analysis were collected at a rate slightly below the incoming flow (2 l/h), while maintaining a higher influx rate of 0.25 l/h to reduce contamination from the surroundings. Roots were enclosed with custom-made cuvettes, (see Danner et al. 2012) fixed with a solvent-free rubber-based sealant (Terostat IX, Henkel, UK). Flows were the same as for the AG plant parts. The differently treated plants were randomly connected to the valves to avoid systemic errors. The temperature in the lab was kept constant at 21 °C throughout the experiments, while a light–dark cycle of 16:8 h was maintained with LED plant lights (6 × 30 W, Green Power LED, Philips, Eindhoven, The Netherlands). PTR-MS data were averaged over the time that each cuvette was sampled each turn (450 sec). As these periods differed slightly between experiments, a moving average with a width of 4 hr was calculated to obtain the same number of sampling time points from each of the experimental replicates.

As PTR-MS is a low-selective method (with standard mass resolution set to unity), a complementary method was used in addition to the on-line detection with the PTR-MS for positive identification of compounds. We confirmed the presence of compounds in the headspace of roots and shoots by collections on thermal desorption tubes filled with 100 mg Tenax TA (60/80 mesh; Supelco, Bellefonte, PA, USA) and subsequent GC/MS analyses following the protocols in (Biesterbos et al. 2014). For reference spectra, see supplementary material (Figure S2 and S3).

### Data Analyses and Statistics

All statistics, data analyses, and graphs were produced using the statistical software package R (R Core Team 2014). The dry weights of the shoots and roots(3 experiments) were checked for normality (Shapiro-test, *P* > 0.05). While shoot dry weights fulfilled the criteria for normality (*P* > 0.05), root dry weights matched only the criteria after log-transformation. ANOVA showed that neither shoot nor root dry weights differed between treatments (*F* = 0.958, *P* > 0.05 and *F* = 2.12, *P* > 0.05, respectively), although they did differ between experimental runs; dry weight differed for both shoot and root tissues (*F* = 12.65, *P* < 0.001 and *F* = 29.85, *P* < 0.001, respectively; Figs. S4, S5). The emission data were standardized with the plant dry weights to correct for differences between experimental runs and differently sized plants. The average of the dry weights for shoot and root tissues over all experiments was used to standardize the signals from the first of the four experiments, for which dry weights were missing. The distribution of damage levels across treatments was investigated with a *Chi*-*Square* test. No differences in damage percentage were found between treatments for either AG or BG organs (*P* = 0.319, *P* = 0.606, respectively).

### Statistical Analysis of Time Series Data

The emission data vary not only in time, but also between repeated experiments. There were not sufficient data points recorded to determine an appropriate model for the entire time series. Therefore, we reduced the data to produce a reasonable model by choosing five time points uniformly spread over the entire time interval at precisely 12, 28, 44, 60, and 76 hr after the start of the experiment. Due to the dynamic range of the data, they were log (1 + x)-transformed to obtain normality. The emission levels at the chosen time points turned out to be strongly correlated. Therefore, we could not use a standard linear model approach. Instead, we modeled 4 (treatments) × 8 (replicates) = 32 vectors describing the dynamics of the emissions over the 5 chosen time points. Using these vectors, we designed a model to detect differences in emissions patterns between treatment groups. We used a simple dependence structure, whereby we assumed that the measurements at our five time points had the same covariance structure as a first order autoregressive time series (Shumway and Stoffer 2011). This means that if X = (X1, X2, …, X5) is the measured vector, then the covariances are given by

$$ \mathrm{C}\mathrm{o}\mathrm{v}\ \left(\mathrm{Xi},\mathrm{X}\mathrm{j}\right)=\upsigma\ 2\uprho\ \left|\mathrm{i}-\mathrm{j}\right| $$

Here, σ > 0 and ρ ∈ [−1, 1] are unknown parameters that were estimated from the data: σ represents the standard deviation of the emission at a time point and ρ represents the correlation between two subsequent time points. After estimating the parameters, we found a strong correlation between time points with *ρ* = 0.8 on average. To this end, we used a one-sided *t*-test, taking into account the special covariance structure of the model. After estimating ρ and σ in the full model, we looked at the difference in the average of the five time points and standardized this difference by dividing it by its own estimated standard deviation (which only depends on ρ and σ). Based on this, we calculated a one-sided p-value for each comparison (each treatment against all others; Table S1).

## Results

We confirmed the positive identification of sulfur compounds monitored on-line with PTR-MS with GC/MS analysis of the same compounds collected from the plant headspace on thermodesorption tubes. Their mass spectra are shown in the supplementary material (Figs. S2, S3). Several volatile organic sulfur compounds [*m/z* 49 – methanethiol, *m/z* 63 – dimethylsulfide (DMS), and *m/z* 95 – dimethyldisulfide (DMDS)] as well as methanol (*m/z* 33) were emitted from both AG and BG plant parts with stronger overall emission rates from belowground tissues in all treatments.

### Aboveground VOCs

**Fig. 2:**
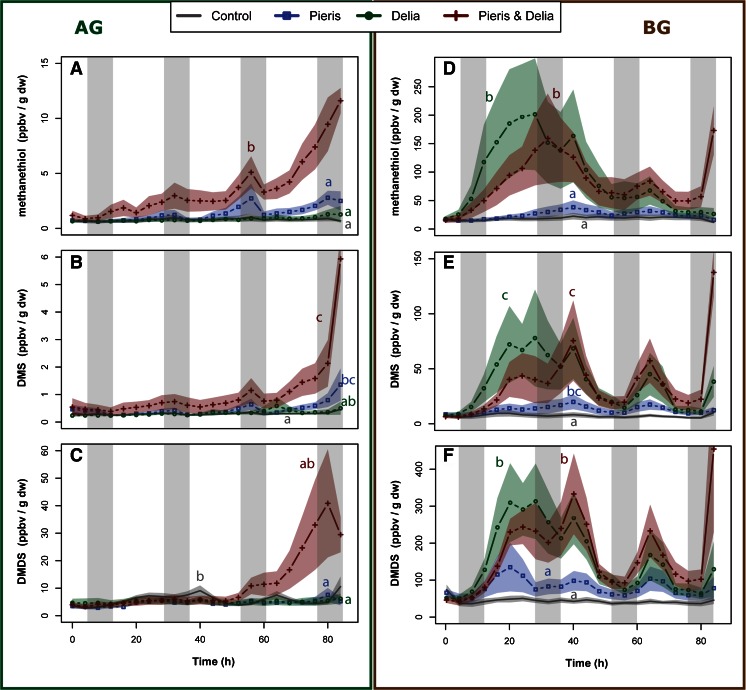
Time-resolved emission of sulfur-containing compounds from *Brassica rapa* shoots aboveground (*AG*, *left*) and roots belowground (*BG*, *right*) infested only with root feeding *Delia radicum* larvae (*green lines plus circles*), leaf feeding *Pieris brassicae* larvae (*blue line plus squares*) or both (*red line plus crosses*). *Grey lines* are undamaged plants. Emission of methanethiol (*m/z* 49; panel **a** and **d**), dimethylsulfide (DMS; *m/z* 63; panel **b** and **e**), and dimethyldisulfide (DMDS; *m/z* 93; panel **c** and **f**) are represented in gas mixing ratios (parts per billion volume) normalized over the dry weight (in gram) of the respective plant organ. *Colored bands* represent the standard errors (+/− 1 SE, *N* = 8). Night periods are indicated by *grey shading. Different letters* indicate the results of the autoregressive time series model (see supplementary Table S1)

**Fig. 3:**
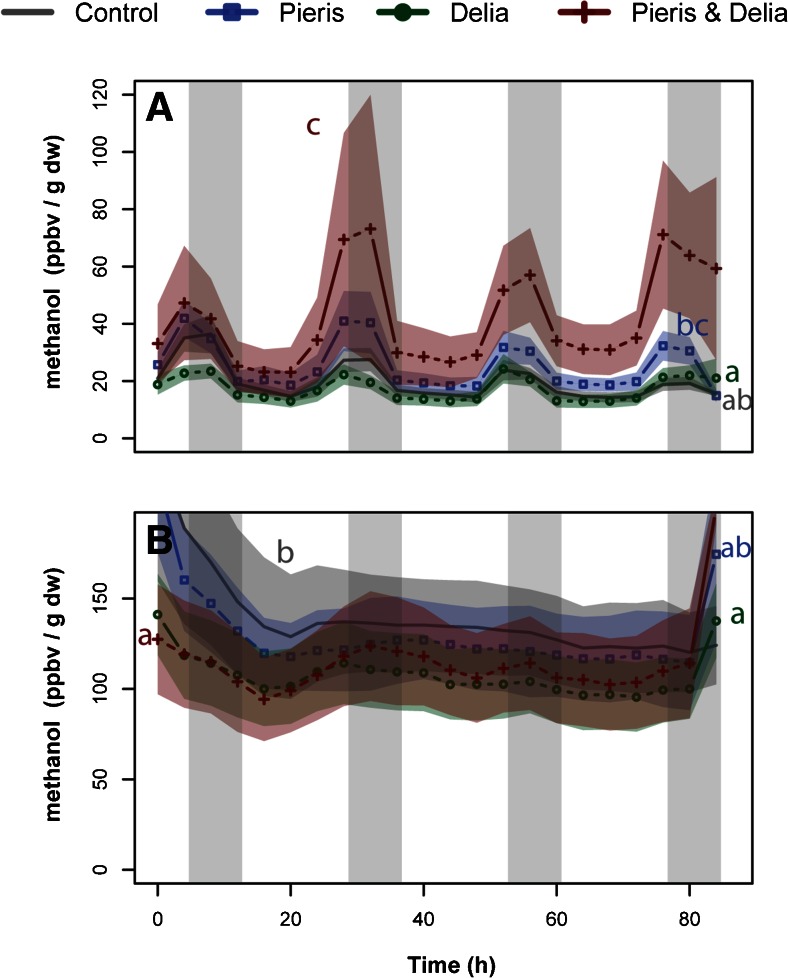
Time-resolved emission of methanol (*m/z* 33) from *Brassica rapa* shoots (**a**, *upper* panel) and roots (**b**, *lower* panel) infested only with root feeding *Delia radicum* larvae (*green lines plus circles*), leaf feeding *Pieris brassicae* larvae (*blue line plus squares*), or both (*red line plus crosses*). *Grey lines* are undamaged plants. Methanol emissions are represented in gas mixing ratios (parts per billion per volume) normalized over the dry weight (in gram) of the respective plant organ. *Colored bands* represent the standard errors (+/− 1 SE, *N* = 8). Night periods are indicated by *grey shading. Different letters* indicate the results of the autoregressive time series model (see supplementary Table S1)

Plants infested with both herbivores emitted methanethiol from their shoots in higher quantities than in any other treatment (Fig. 2a, *P* < 0.01). Methanethiol emissions of shoot-infested plants were induced only slightly and were not statistically different from control plants (*P* = 0.063). When both herbivores were feeding simultaneously, DMS emissions from the shoots were increased compared to controls and root-infested plants (Fig. 2b, *P* < 0.05), whereas they were not significantly different from shoot-infested plants. Plants infested only on their shoots still emitted more DMS than controls (Fig. 2b, *P* < 0.05). The DMDS emissions from double-infested plants were enhanced overall, but did not differ statistically from shoot-induced or root-induced plants (Fig. 2c, *P* = 0.066 and *P* = 0.068, respectively). DMDS emissions of control plants were significantly different over the course of the experiment from those of shoot and root-infested plants; at several time points, the emissions of DMDS from controls exceeded that of root or shoot infested plants (Fig. 2c, *P* < 0.05). Emission of methanol from AG tissues showed a diurnal rhythm in all treatments (Fig. 3a) with maxima during the night. Emissions were highest in double-infested plants, which emitted more methanol than controls and root-infested plants (Fig. 3a, *P* < 0.01). Additionally, *P. brassicae* infested plants emitted more methanol than root-infested plants (*P* < 0.01), but not significantly more than control plants (Fig. 3a, *P* = 0.074).

### Temporal Dynamics of Aboveground Sulfur VOC Emissions

*Pieris brassicae* infestation increased local AG emissions of methanethiol and DMS from shoots at about 55 hr after infestation (Fig. 2a, b), whereas emission of DMDS remained at the level of the controls throughout the measurements (Fig. 2c). Double-infested plants already started to emit higher amounts of DMS and DMDS than controls, around 20 hr post infestation, with another emission peak for all three compounds at 60 hr post infestation. This increase continued until the end of the measurements. All sulfur compounds responded similarly when plants were infested with both herbivores, but DMDS emissions initially did not differ as strongly from the other treatments as DMS and methanethiol. Root infestation with *Delia radicum* alone had minor effects on the systemic emission of sulfides from shoots; methanethiol and DMS emission increased only slightly and not significantly over control levels.

### Belowground VOCs

Root-infested and double-infested plants emitted higher amounts of methanethiol than controls (*P* < 0.001) and shoot-infested plants (Fig. 2d, *P* < 0.05). Shoot infestation also appeared to increase methanethiol emissions, but not significantly (Fig. 2d, *P* = 0.069). Emissions of DMS and DMDS showed the same pattern globally as methanethiol (Fig. 2d–f). For DMS (Fig. 2e), shoot-induced plants also emitted higher levels of DMS than controls (*P* < 0.05). After root infestation and double infestation, DMDS was emitted in higher amounts compared to controls (Fig. 2f, *P* < 0.001), and shoot-infested plants (*P* < 0.01). Emission of DMDS was enhanced overall in dual infested plants compared to single infestations (*P* = 0.051). Methanol emissions from roots did not show a diurnal pattern (Fig. 3b), but control plants emitted higher amounts of methanol compared to root-infested and double infested plants (*P* < 0.05).

### Temporal Dynamics of Sulfur Compound Emissions Belowground

The temporal pattern of roots was completely different from the temporal pattern of shoots. At most time points, plants with only root infestation emitted similar amounts of sulfur-containing VOCs from roots as plants infested by both herbivores (Fig. 2d–f). Furthermore, the first emission maximum for all sulfur compounds appeared simultaneously at around 20 hr post infestation. Moreover, there was a second emission peak at around 65 hr and a final increase at the end of the measurement period that was steeper for plants infested with both herbivores. Additionally, we found a slight systemic increase in sulfur compounds after shoot infestation with *P. brassicae*, which did not occur in shoots of plants infested with root fly larvae only.

## Discussion

In this study, we showed that shoots of plants infested with both AG and BG herbivores emitted higher amounts of sulfur-containing compounds than those with a single herbivore. With the exception of DMS, AG herbivory alone did not significantly increase sulfur VOC emissions over control levels. There were no systemic effects of root herbivore feeding on shoot sulfur VOC emissions. Thus, the interactive effects of root and shoot herbivores are a prerequisite for enhanced emissions of sulfur-containing compounds from AG tissues. In BG tissues, infestation with root herbivores alone was sufficient to induce fast and strong local emissions of all three sulfur-containing compounds: the addition of a shoot herbivore to the same plant did not further increase belowground-induced sulfur VOC emissions. Shoot herbivory alone caused a slight, but significant systemic response in DMS emissions from roots. Overall, roots emitted higher amounts of sulfur-containing VOCs per gram dry mass than shoots. Moreover, the local response to herbivory with regards to sulfur VOC emissions was faster and showed a more dynamic pattern over time in roots than in shoots. In addition, we found that methanol emissions from the shoots showed clear diurnal patterns, whereas root emissions did not. In shoots, methanol emissions were increased by AG herbivory as well as by double herbivory, whereas root emissions from infested plants generally decreased.

Interactive effects of herbivores on plant VOC emissions have been described previously for AG plant parts under attack by multiple species (Dicke et al. 2009). Our results add to the evidence that such interactions also occur between root and shoot feeding herbivores. However, for sulfur-containing VOCs emitted from double infested *B. rapa* plants, the observed AG-BG interactive effects are not symmetrical. Shoot emissions were clearly enhanced by the presence of root feeders on the same plant, whereas locally induced emissions from roots were not further increased by an additional herbivore aboveground. The first result supports the hypothesis that sulfur-containing compounds can serve as a reliable cue to *C. glomerata* wasps, indicating the presence of root herbivores on a plant where their preferred host, *P. brassicae*, is feeding (Soler et al. 2007). Moreover, the data show that differences in shoot emissions alone may be sufficient for these wasps to learn to distinguish the difference between single or double infested plants (Kruidhof et al. 2013); diffusion of BG produced sulfides into the AG headspace, if at all, would not provide additional cues. This does not preclude that changes in the emissions of other VOCs, such as terpenes, whose emissions decrease in root-induced plants, add to the ability of the wasps to discriminate between plants with different combinations of root and shoot herbivores (Soler et al. 2007; van Dam et al. 2010). The same differences in VOC profiles can be used by the *D. radicum* parasitoid, *T. rapae*. It has been shown previously that this parasitoid, while hunting for a BG host, specifically responds to volatile cues from AG plant parts (Neveu et al. 2002; Pierre et al. 2011a). Similar to *C. glomerata*, which prefer plants infested only with their host over plants infested also on the roots, the BG parasitoid *T. rapae* prefers plants without herbivores feeding on the shoots simultaneously with the roots. (Pierre et al. 2011a). As root herbivory alone did not increase emissions of sulfur-containing compounds over control levels, these parasitoids most likely use information carried by other classes of VOCs, such as 4-methyltridecane, to locate plants infested with *Delia* larvae only (Pierre et al. 2011b). Detection of long chain alkanes, such as 4-methyltridecane was observed with PTR-MS by using different optimum settings from those used herein, suggesting that detecting this class of VOC from plant sources also may be possible with PTR-MS (Erickson et al. 2014).

The lack of response in AG tissues after shoot herbivory may be attributed to our focus on sulfur-containing compounds. DMDS has been found previously to decrease after *P. brassicae* feeding on two different cabbage cultivars (Geervliet et al. 1997). This and other studies also have shown that other VOCs, such as terpenes and acetates, may be induced more strongly than sulfur compounds by caterpillar feeding on *Brassica* plants (Pierre et al. 2011b).

Other than for shoot emissions, sulfur VOC emissions from *B. rapa* roots were not further enhanced by the presence of an AG herbivore, despite the fact that AG herbivory alone significantly induced BG DMS emissions. This is in contrast with other studies where AG herbivory often reduces the emissions of BG induced volatiles. For example, in corn infested with the root herbivore *Diabrotica virgifera*, *E*-β-caryophyllene content from the roots decreased when a shoot herbivore was feeding simultaneously (Rasmann and Turlings 2007). Similarly, grass hybrids showed reduced emissions of terpenes, acetic acid, and C6 compounds when infested with an AG endophyte (Rostas et al. 2015). The ecological role of sulfide emissions in BG multitrophic interactions is more poorly studied than that of root emitted terpenes (Penuelas et al. 2014). For DMDS, it is known that ground dwelling predators such as staphylinid beetles effectively respond to this compound to locate root fly eggs and larvae (Ferry et al. 2007). On the other hand, adult female root flies themselves are deterred by high levels of DMDS (Ferry et al. 2009), which prevents them ovipositing on plants already heavily infested by competitors for their offspring. Whether methanethiol, DMS, or DMDS is specifically well-suited for BG signaling, as has been shown for certain glucosinolate breakdown products (Matthiessen and Kirkegaard 2006) and E-β-caryophyllene emitted by maize roots (Rasmann et al. 2005) needs to be investigated.

Interestingly, the emissions of roots and shoots follow independent temporal patterns; sulfide emissions from roots increased much faster upon local herbivory than in shoots. This may be explained by the fact that both larval feeding as well as mechanical wounding may increase the rapid emission of methanethiol, DMS, and DMDS from the shoots of *B. rapa* (van Dam et al. 2012). Similar information for the induction of sulfides in shoots is missing, but the fact that sulfide emissions AG increased much later suggests that their production in the shoot may require the expression of specific genes, such as thiomethyl transferase (TMT) or cysteine-S-lyase (Attieh et al. 2000; Chin and Lindsay 1994). Whether these and other genes, e.g., involved in the different signaling pathways, are differentially expressed under single and dual herbivory should be elucidated by transcriptome analyses.

Both shoots and roots were found to emit methanol, but the observed response to herbivory was herbivore dependent. Similar to what has been reported from other plant species (von Dahl et al. 2006; Penuelas et al. 2005), shoot damage increased methanol emissions from *B. rapa* shoots. However, this increase was much stronger in double infested plants, suggesting that enzymes involved in methanol production must be more strongly activated when there is simultaneous root feeding (Körner et al. 2009). Root damage alone did not result in significantly increased methanol emissions from the shoots, indicating that the enhanced emissions in double infested plants result from cross-talk between differently induced signaling pathways. Root emissions generally decreased upon herbivory, independent on where the herbivore was feeding. It is known that roots contain the gene coding for pectin methylesterase (PME) responsible for the production of methanol (Oikawa et al. 2011). It is not known if the regulation of this gene is contingent on the organ in which it is activated and we propose that given our results, this should be tested. The exact biological role of methanol is not well known. It may either serve as an induction signal triggering defense responses in plants (von Dahl et al. 2006) or provide broad spectrum insect resistance (Dixit et al. 2013).

In summary, interactive effects of root and shoot herbivores mainly affect the emissions of sulfur-containing volatiles and methanol from the shoots and not from the roots. This implies that there are specific interactions on the level of signaling hormones that determine shoot VOC emissions. The nature of these interactions is currently being analyzed in more detail on the transcriptome and phytohormone level.

## Electronic supplementary material

Table S1 Results of *t*-test from the first order autoregressive time series model. CC – controls, HC – *Delia radicum* infested plants, CH – *Pieris brassicae* infested plants, HH – plants infested with *D.radicum* + *P. brassicae*. (GIF 177 kb)

High Resolution Image (TIFF 5745 kb)

FIG. S2 Mass Spectrum of methanethiol from the plant head space, collected on thermodesorption tubes (200 mg Tenax) and GC-MS analysis with NIST reference spectrum (red) (GIF 21 kb)

High Resolution Image (TIFF 373 kb)

FIG. S3 Mass Spectrum of DMS and DMDS from the plant head space, collected on thermodesorption tubes (200 mg Tenax) and GC-MS analysis with NIST reference spectrum (red) (GIF 46 kb)

High Resolution Image (TIFF 528 kb)

FIG. S4 Means of aboveground dry weights per experiment and per treatment. Results of Anova statistics: between experiments (*F* = 12.65, *P* < 0.001) and between treatments (*F* = 0.958, *P* > 0.05). (GIF 42 kb)

High Resolution Image (TIFF 488 kb)

FIG. S5 Means of belowground dry weights per experiment and per treatment. Results of Anova statistics: between experiments (*F* = 29.85, *P* < 0.001) and between treatments (*F* = 2.12, *P* > 0.05). (GIF 42 kb)

High Resolution Image (TIFF 503 kb)
