# Supplementary figures and images for: Aboveground and Belowground Herbivores Synergistically Induce Volatile Organic Sulfur Compound Emissions from Shoots but Not from Roots
Source: J Chem Ecol. 2015 Jul 21;41(7):631–40. doi: 10.1007/s10886-015-0601-y (PMC4525197; doi:10.1007/s10886-015-0601-y)

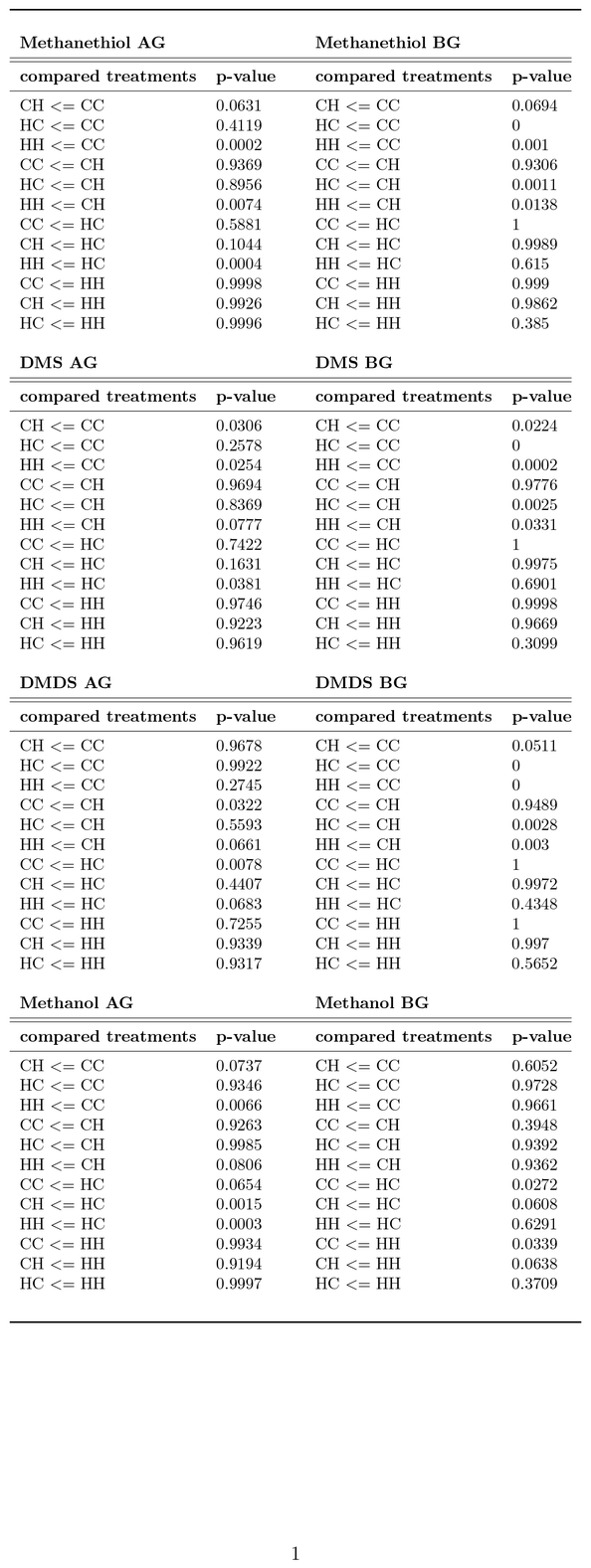

Supplement: Supplementary file 1 — Results of t-test from the first order autoregressive time series model. CC – controls, HC – Delia radicum infested plants, CH – Pieris brassicae infested plants, HH – plants infested with D.radicum + P. brassicae. (GIF 177 kb) [file 10886_2015_601_Fig4_ESM.gif]

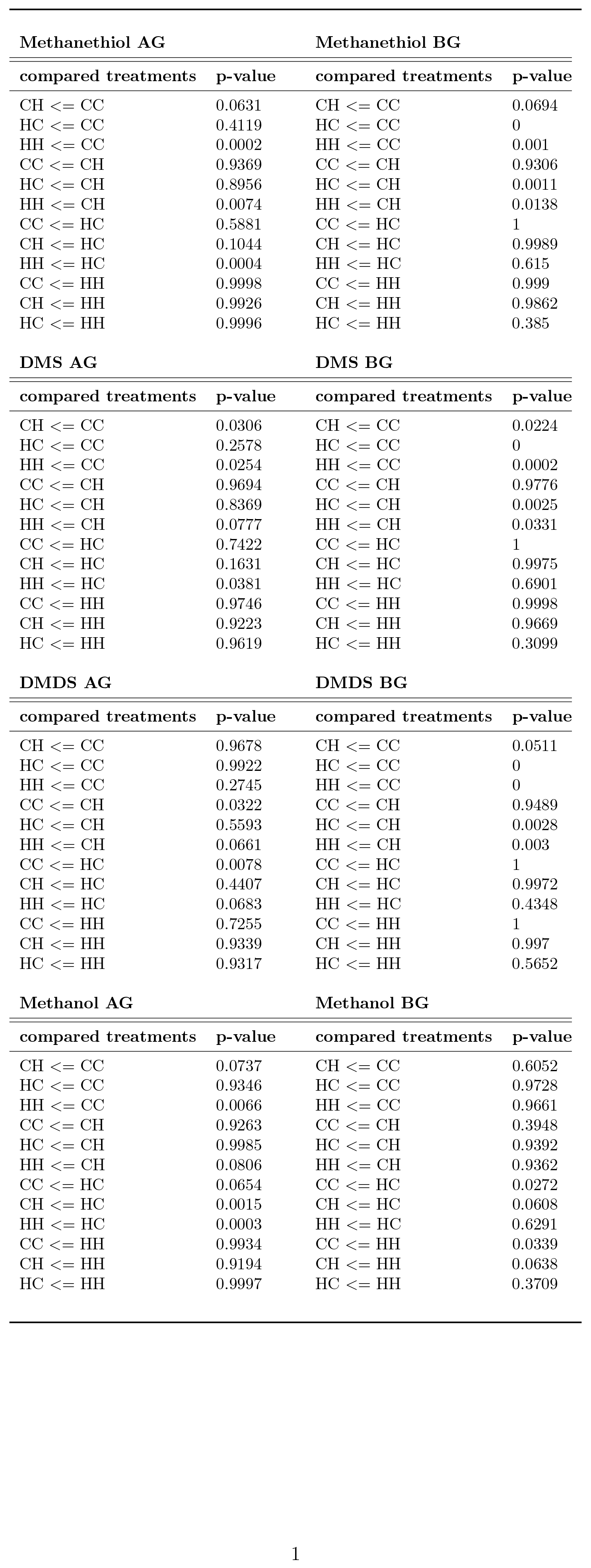

Supplement: Supplementary file 2 — High Resolution Image (TIFF 5745 kb) [file 10886_2015_601_MOESM1_ESM.tif]

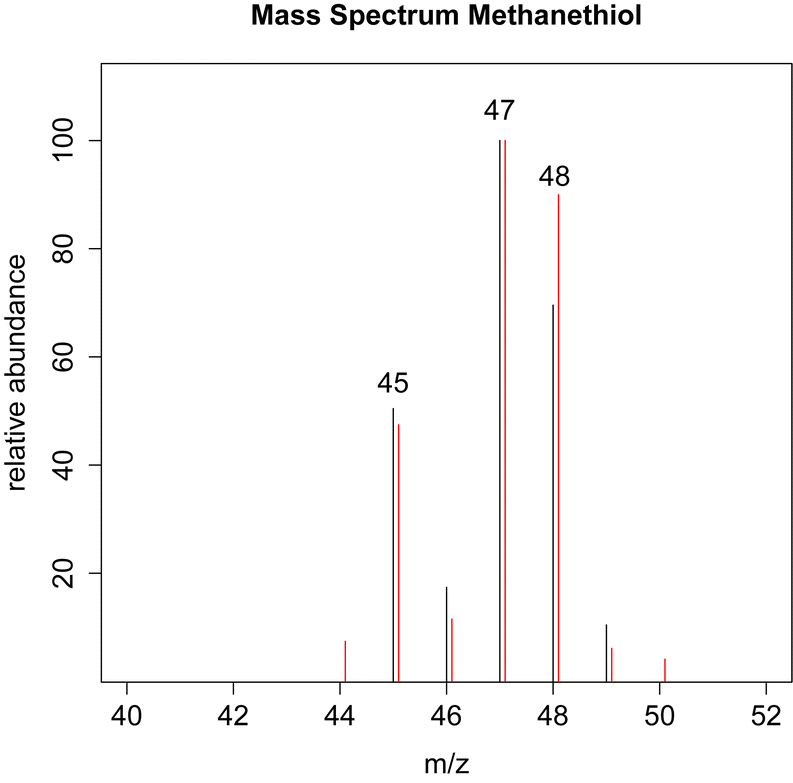

Supplement: Supplementary file 3 — Mass Spectrum of methanethiol from the plant head space, collected on thermodesorption tubes (200 mg Tenax) and GC-MS analysis with NIST reference spectrum (red) (GIF 21 kb) [file 10886_2015_601_Fig5_ESM.gif]

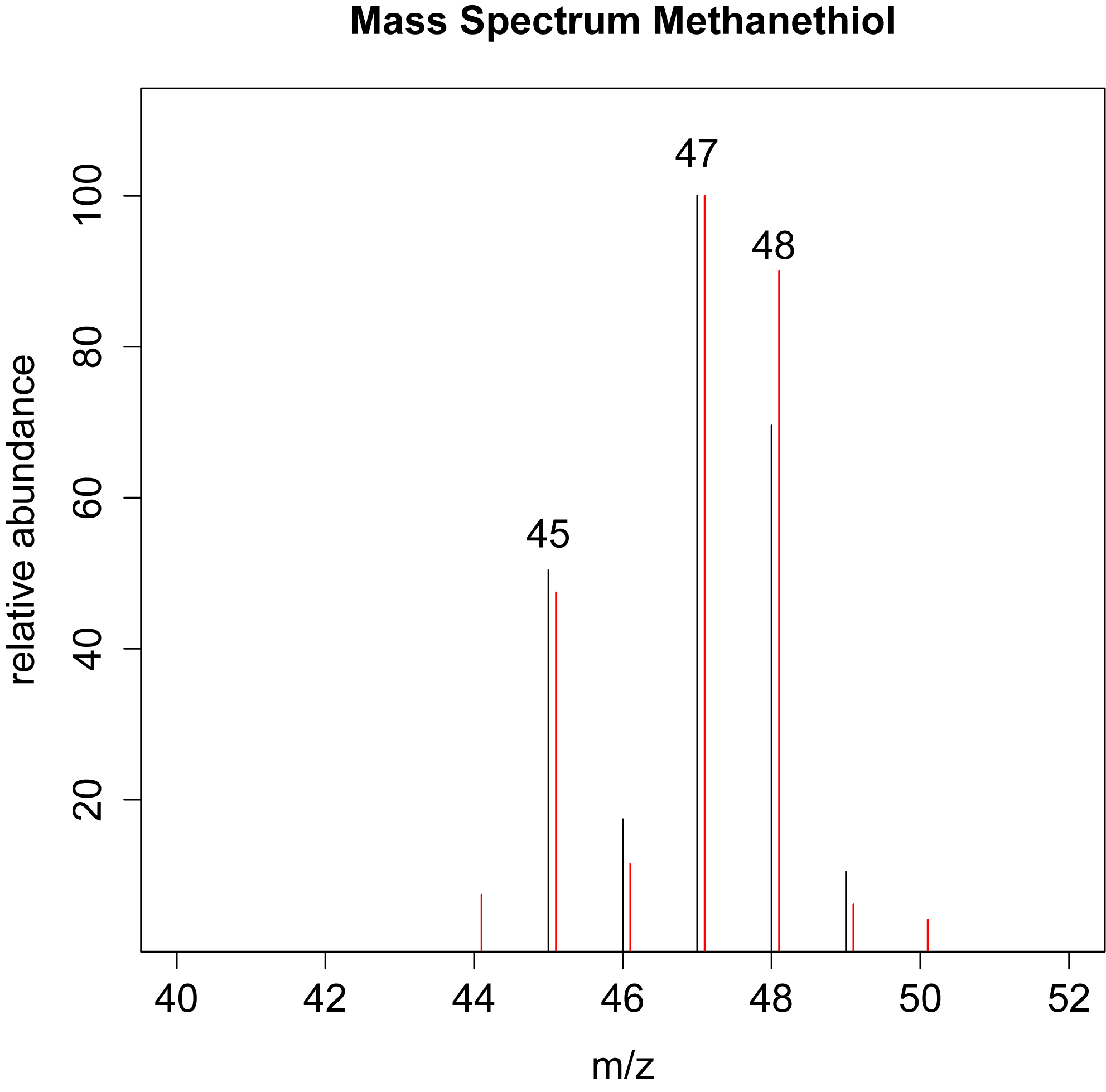

Supplement: Supplementary file 4 — High Resolution Image (TIFF 373 kb) [file 10886_2015_601_MOESM2_ESM.tif]

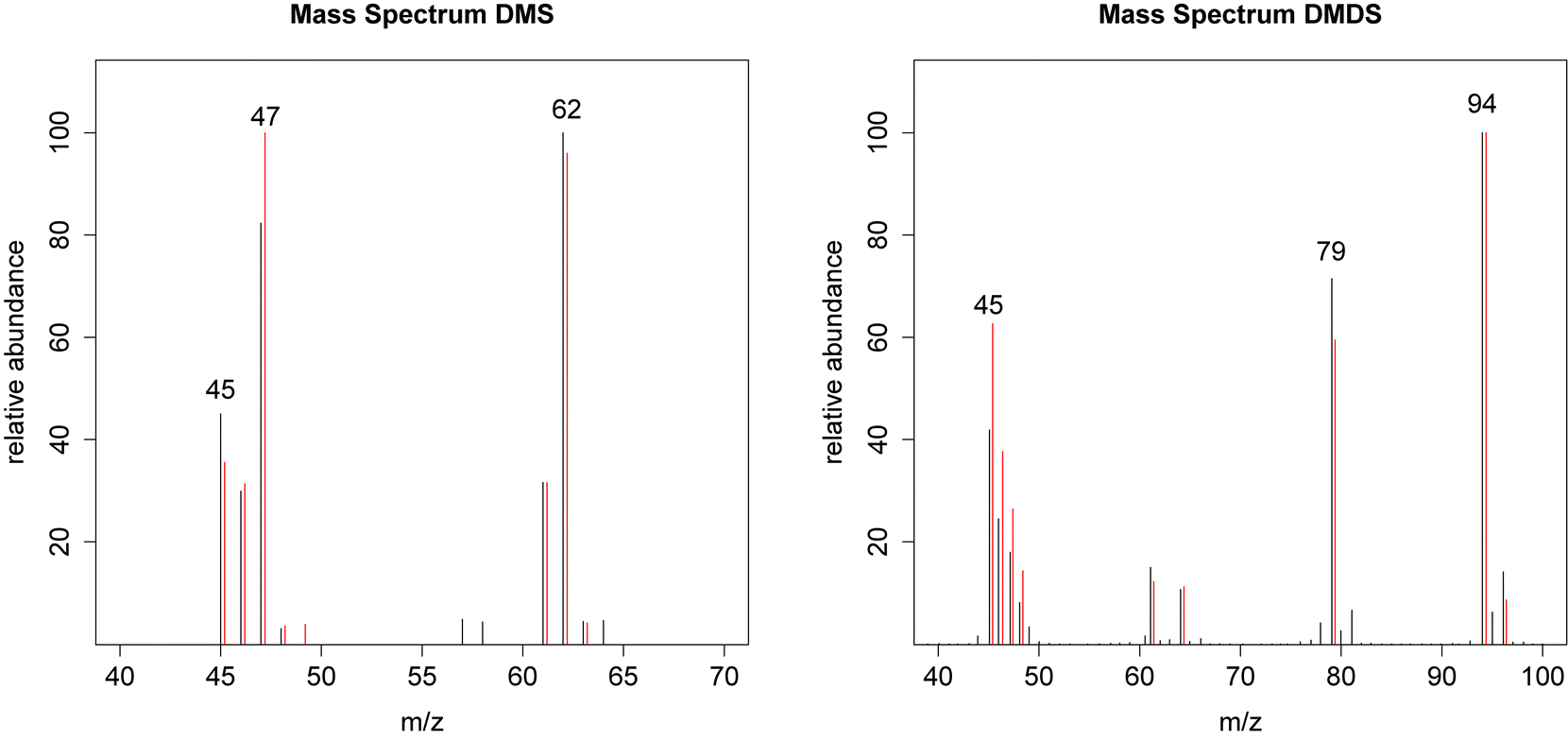

Supplement: Supplementary file 5 — Mass Spectrum of DMS and DMDS from the plant head space, collected on thermodesorption tubes (200 mg Tenax) and GC-MS analysis with NIST reference spectrum (red) (GIF 46 kb) [file 10886_2015_601_Fig6_ESM.gif]

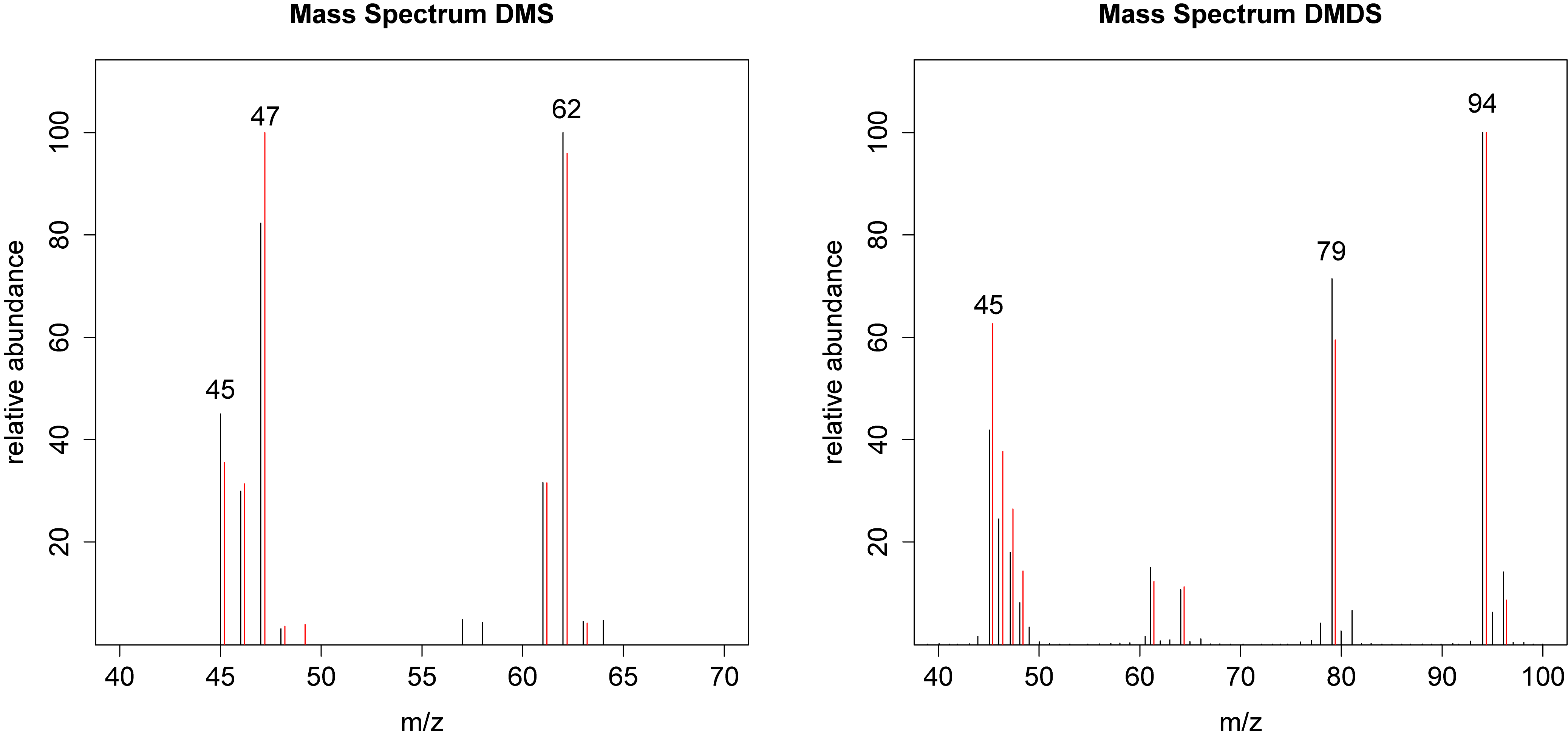

Supplement: Supplementary file 6 — High Resolution Image (TIFF 528 kb) [file 10886_2015_601_MOESM3_ESM.tif]

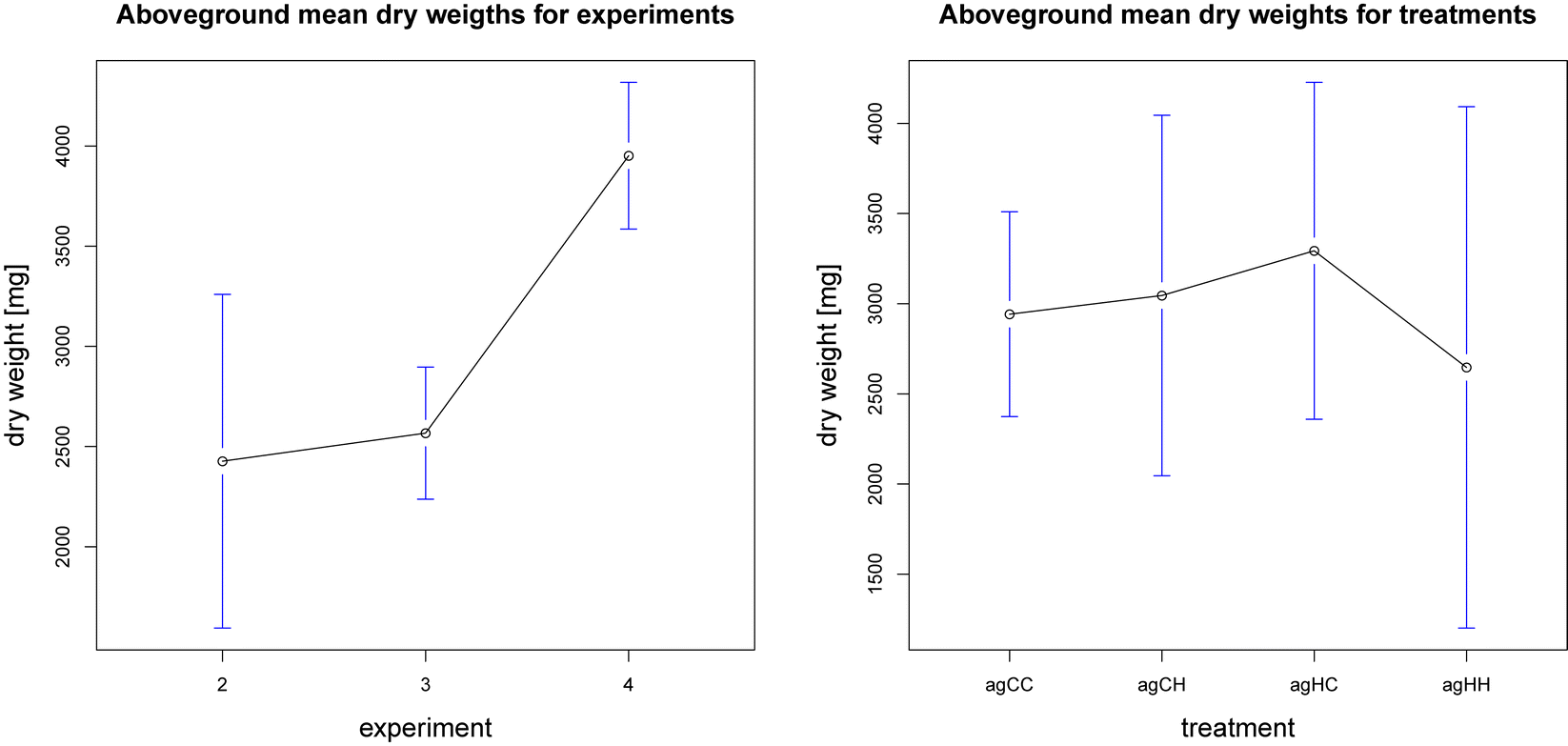

Supplement: Supplementary file 7 — Means of aboveground dry weights per experiment and per treatment. Results of Anova statistics: between experiments (F = 12.65, P < 0.001) and between treatments (F = 0.958, P > 0.05). (GIF 42 kb) [file 10886_2015_601_Fig7_ESM.gif]

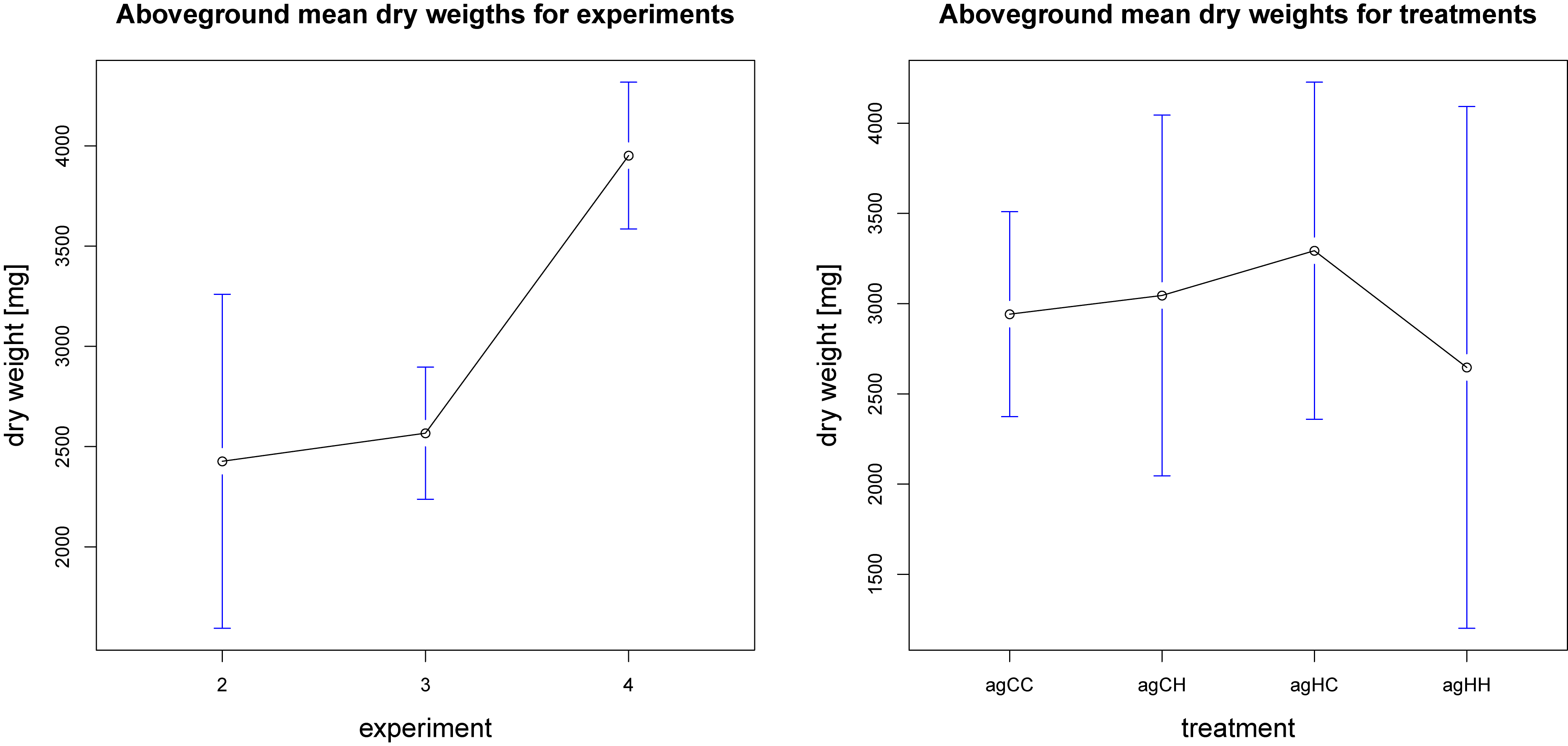

Supplement: Supplementary file 8 — High Resolution Image (TIFF 488 kb) [file 10886_2015_601_MOESM4_ESM.tif]

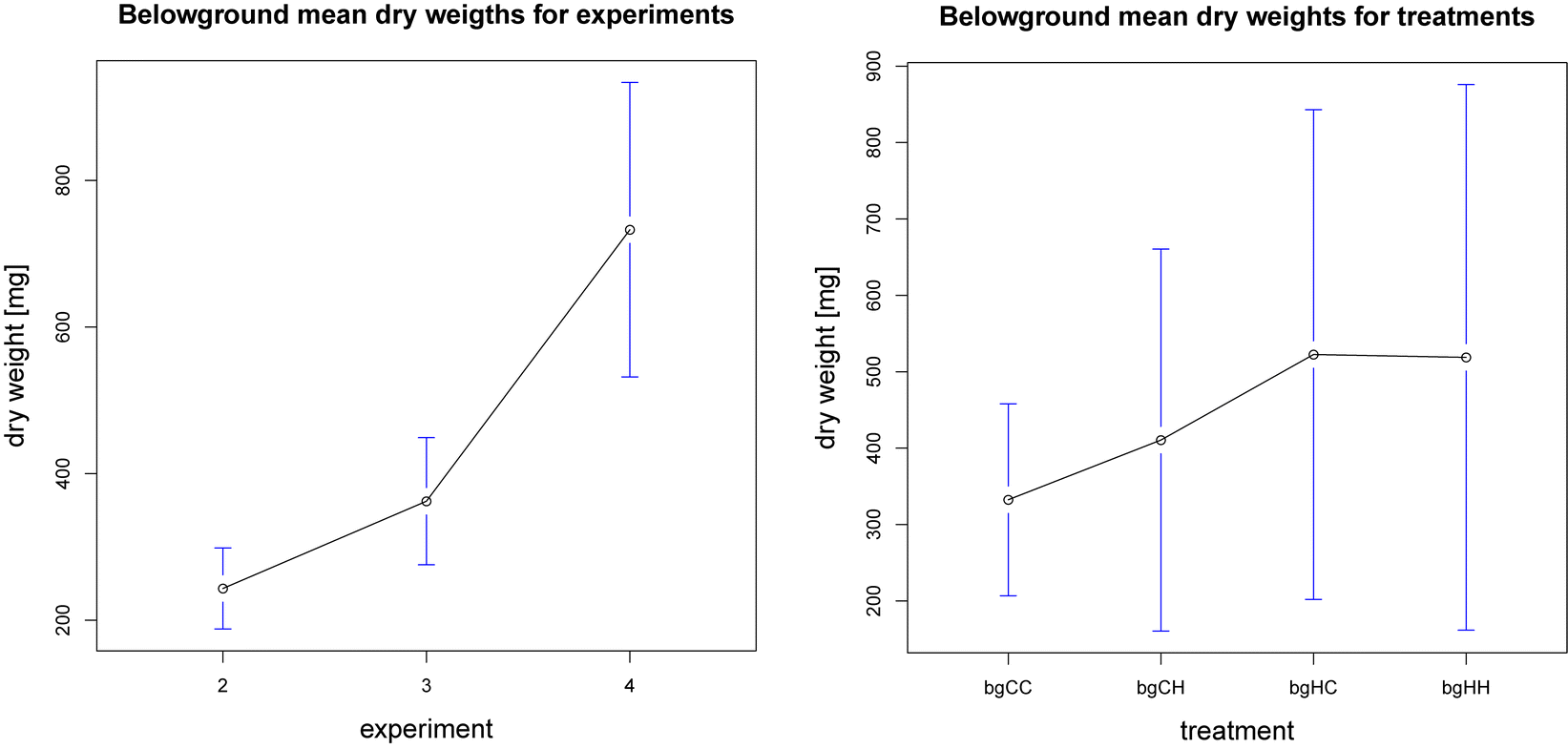

Supplement: Supplementary file 9 — Means of belowground dry weights per experiment and per treatment. Results of Anova statistics: between experiments (F = 29.85, P < 0.001) and between treatments (F = 2.12, P > 0.05). (GIF 42 kb) [file 10886_2015_601_Fig8_ESM.gif]

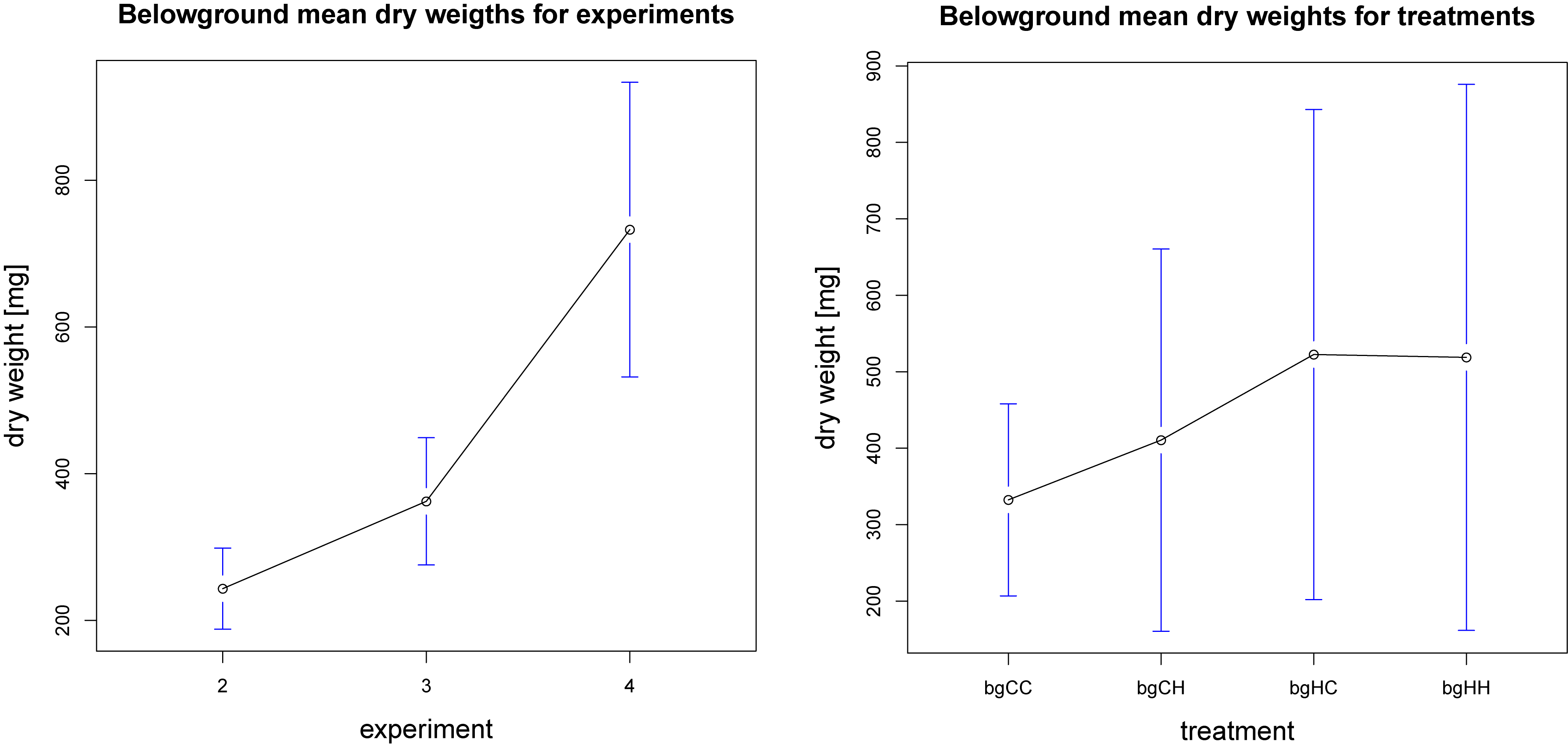

Supplement: Supplementary file 10 — High Resolution Image (TIFF 503 kb) [file 10886_2015_601_MOESM5_ESM.tif]
